# To Our Friends and Correspondents

**Published:** 1880-04

**Authors:** 


TO OUR FRIENDS AND CORRESPONDENTS.—We hereby tender our esteemed friends and correspondents, one and all, our most sincere thanks for their warm commendations of our editorial utterances. It is always pleasant to learn that our efforts are appreciated by those for whom we labor. But we had not thought to receive such words of cheer and encouragement for what we have thus far said, as we have striven to carry out the objects announced in our prospectus only. We shall still hope to merit the approval, as far as possible, of the honorable and intelligent in our profession by continuing to uphold the right, and to deprecate, and even to denounce wrongdoing as occasion might require, and that too without fear, favor or affection.

